# Cutaneous Melanoma: Mutational Status and Potential Links to Tertiary Lymphoid Structure Formation

**DOI:** 10.3389/fimmu.2021.629519

**Published:** 2021-03-04

**Authors:** Deepak Salem, Manoj Chelvanambi, Walter J. Storkus, Ronald J. Fecek

**Affiliations:** ^1^Department of Microbiology, Lake Erie College of Osteopathic Medicine at Seton Hill, Greensburg, PA, United States; ^2^Department of Immunology, University of Pittsburgh School of Medicine, Pittsburgh, PA, United States; ^3^Department of Dermatology, University of Pittsburgh School of Medicine, Pittsburgh, PA, United States; ^4^Department of Pathology, University of Pittsburgh School of Medicine, Pittsburgh, PA, United States; ^5^Department of Bioengineering, University of Pittsburgh School of Medicine, Pittsburgh, PA, United States

**Keywords:** tertiary lymphoid structures, cutaneous melanoma, tumor mutational burden, driver mutations, DNA repair proteins

## Abstract

Recent advances in immunotherapy have enabled rapid evolution of novel interventional approaches designed to reinvigorate and expand patient immune responses against cancer. An emerging approach in cancer immunology involves the conditional induction of tertiary lymphoid structures (TLS), which are non-encapsulated ectopic lymphoid structures forming at sites of chronic, pathologic inflammation. Cutaneous melanoma (CM), a highly-immunogenic form of solid cancer, continues to rise in both incidence and mortality rate, with recent reports supporting a positive correlation between the presence of TLS in melanoma and beneficial treatment outcomes amongst advanced-stage patients. In this context, TLS in CM are postulated to serve as dynamic centers for the initiation of robust anti-tumor responses within affected regions of active disease. Given their potential importance to patient outcome, significant effort has been recently devoted to gaining a better understanding of TLS neogenesis and the influence these lymphoid organs exert within the tumor microenvironment. Here, we briefly review TLS structure, function, and response to treatment in the setting of CM. To uncover potential tumor-intrinsic mechanisms that regulate TLS formation, we have taken the novel perspective of evaluating TLS induction in melanomas impacted by common driver mutations in BRAF, PTEN, NRAS, KIT, PRDM1, and MITF. Through analysis of The Cancer Genome Atlas (TCGA), we show expression of DNA repair proteins (DRPs) including BRCA1, PAXIP, ERCC1, ERCC2, ERCC3, MSH2, and PMS2 to be negatively correlated with expression of pro-TLS genes, suggesting DRP loss may favor TLS development in support of improved patient outcome and patient response to interventional immunotherapy.

## Introduction

Cutaneous melanoma (CM) is a deadly cancer that arises from molecular alterations in melanocytes, the pigment producing cells of the skin (1). Though CM accounts for <5% of all skin cancer cases, it accounts for the most deaths (2). Over the past 30 years, the incidence of CM has steadily increased and in 2020, there will be an estimated 100,350 new cases of CM with an estimated 6,850 deaths related to this disease (3, 4). The identification and presence of tumor infiltrating lymphocytes (TIL) in CM correlates with improved prognosis, supporting assertions that CM is an immunogenic cancer (5, 6). One reason for the strong immune system response to CM is believed to reflect it having one of the highest rates of tumor mutational burden (TMB) amongst solid cancers (7–9). The high TMB results in the generation of novel mutated neoantigens, which have not been subjected to central tolerance mechanisms shaping the repertoire of the adaptive immune system (9). Hence, tumors expressing high numbers of neoantigens tend to elicit more robust anti-tumor T cell responses in concert with a pro-inflammatory TME (10).

The presence of inflammation and particularly CD8^+^ effector T cells in CM may predict a favorable clinical response to immune checkpoint inhibitors (ICI), additionally reinforcing the important role the immune system plays in melanoma (11). Furthermore, because of their clinical efficacy, ICI such as ipilimumab (anti-cytotoxic T-lymphocyte-associated protein-4 (CTLA-4) monoclonal antibody (mAb)), pembrolizumab, and nivolumab (both anti-programmed death-1 (PD-1) mAbs) when applied as monotherapies or combinational therapies have dominated recent immunotherapeutic clinical trial designs and are expected to continue to do so for many years to come (12–14). Nevertheless, most patients treated with these advanced immunotherapeutic agents exhibit intrinsic or acquired resistance to treatment (12, 15), emphasizing the need for an improved understanding of the dynamic interactions between the immune system and CM in the TME for the design of improved interventional approaches.

## Tertiary Lymphoid Structures in Brief

An exciting new paradigm in cancer immunology involves the role of TLS as regulators of disease progression and effective immunotherapy (16). TLS are ectopic lymphoid organs which are similar in function to secondary lymphoid organs (SLO), such as lymph nodes, but they form in areas of the body that do not normally house lymphoid tissue (17, 18). In this regard, SLO are considered “hard-wired” in forming at developmentally-programmed anatomic locations, while TLS evolve adaptively, forming in peripheral tissue sites impacted by chronic inflammation. Development of TLS in affected tissues is believed to be initiated by a complex crosstalk between recruited inflammatory leukocytes, local stromal cells, and a local source of sustained tissue insult (i.e., unresolved pathogens, infected cells, or mutated cells) (19).

TLS consist of organized aggregates of T cells, B cells, dendritic cells (DC), follicular dendritic cells (FDC), T follicular helper (Tfh) cells, specialized stromal fibroblasts, and high endothelial venules (HEV) (20, 21). When compared with SLO, TLS are non-encapsulated which is thought to facilitate rapid antigen presentation at the site of inflammation and, in the context of cancer, allows the locally primed immune system to quickly mediate surveillance of cognate tumor-associated (neo)antigens (18). Classic mature TLS exhibit compartmentalized zones for immune specialization, such as B cell follicles surrounding germinal centers (GC) containing rapidly proliferating/differentiating B cells (producing antibodies) and distinct T cell zones enriched in conventional DC-LAMP^+^ DC, akin to those found in SLO (22, 23). Non-classic TLS which are deficient in B cells/GC and composed largely of T cells and DC clustered around HEV have also been described in human cancers (24). Remarkably, both classic and non-classic TLS have been reported as prognostic biomarkers of improved clinical outcome amongst cancer patients (25). It is also important to note that TLS cellular composition is highly-variable over time, being impacted by local alterations in newly-recruited immune infiltrates and cytokine/chemokine profiles in the progressive/therapeutic TME (26–28).

Lymphangiogenesis is also strongly associated with TLS formation and the formation of HEVs that express peripheral node addressins (PNAd), a ligand for L-selectin, along with CCR7-ligand chemokine CCL21, characteristic of TLS formation vs. simple acute local inflammation (28, 29). Expression of CCL19, CCL21, ICAM-1, and MAdCAM on TLS-associated HEVs enables extravasation of circulating naïve CCR7^+^ T cells, as well as, CCR7^+^ DC into the TME (28). In a complementary fashion, expression of TLS-derived CXCL13 recruits naïve CXCR5^+^ B cells into the TME (18, 29). Once recruited, naïve B and T cells are exposed to cancer (neo)antigens presented by dendritic cell populations, allowing them to differentiate into antibody-producing plasma cells or effector T cells, respectively (30–33). Like conventional DC, memory B cells in classical TLS can serves as effective (neo)antigen-presenting cells to naïve and memory anti-tumor CD8^+^ T cells (32, 33).

TLS have a prognostic, albeit dichotomous, value – in cancer, TLS are generally considered beneficial to the mounting of productive anti-tumor T and B cell responses, but in auto-immune disease such as rheumatoid arthritis, TLS potentiate the immune mediated attack on normal cells where they are considered detrimental (16–18). Furthermore, not all TLS are created operationally equal, and the composition of TLS-component immune subsets must be considered in interpreting impact on local disease pathology. Hence, the presence of regulatory immune cells, such as T regulatory cells (Treg) and myeloid-derived suppressor cells (MDSC) in TLS impacts immunobiologic output and disease severity (26). Although in the autoimmune setting, dominance of suppressor cell populations in TLS ameliorates disease severity and represents a preferred endpoint for treatment intervention (34) in cancer patients such immune deviation is associated with immune tolerance, tumor immune escape and disease progression (35, 36).

## TLS Neogenesis

The generation of TLS shares many similarities with SLO generation, including the presence of complex interactions between mesenchymal lymphoid tissue stromal organizer (LTo) cells and hematopoietic lymphoid tissue inducer (LTi) cells (16, 37). LTβR^+^ (Lymphotoxin Beta receptor), PDPN^+^(podoplanin^+^), LTo cells associated with chronic or acute pathologically inflamed tissue express chemokines and adhesion molecules to attract hematopoietic cells locally (38, 39). Here, TLS neogenesis is initiated with the production of CXCL13, an important mediator of lymphoneogenesis (40). CXCL13, when secreted by activated DCs, stromal cells, and TGF-β stimulated CD8^+^ T cells, recruits and upregulates lymphotoxin (LTα_1_β_2_) on LTi cells (41–44). In CM, CXCL13 has been shown to be over-expressed in primary cells and metastases (45). In addition to CXCL13, IL-7 secretion from stromal cells is another important inducer of lymphotoxin expression on LTi cells (46). Lymphotoxin signaling between $\text{LT}\alpha_{1}\beta_{2}^{+}$ LTi cells, and LTβR^+^ LTo stromal cells is a critical step leading to the upregulation of pro-lymphangiogenic factors, such as VEGF-C, CXCL12, CXCL13, CCL19, and CCL21 (16, 38). The expression of CXCL13, CCL19, and CCL21 from TLS associated stromal cells following lymphotoxin signaling recruits naïve lymphocytes and synergizes with the upregulation of stromal cell/vascular adhesion molecules, facilitating TLS organization (37, 47, 48).

Secretion of these homeostatic chemokines from stromal cells, DCs, and other cells acting as functional LTo cells is important in TLS formation. Activated DCs have been investigated as strong secretors of pro-inflammatory cytokines, such as LTα and LIGHT (aka TNFSF14) (48). Furthermore, B cells in TLS-associated GC have also been reported to serve as strong producers of LIGHT, which is believed to sustain TLS durability *in situ* (49).

VEGF-C is another essential mediator of TLS development (17, 28) and a promoter of lymphangiogenesis (11). It is secreted from activated stromal cells and is a potent inducer of HEV formation (50). HEV formation following VEGF-C stimulation and further HEV-DC lymphotoxin signaling allows for the recruitment of lymphocytes from the circulating blood stream (51). VEGF-C secretion can be induced by IL-6, another pro-inflammatory cytokine secreted by activated DCs (52, 53).

The importance of stromal cells in TLS neogenesis is again highlighted by IL-17 signaling induced by Th_17_ cells (54, 55). Th_17_ cells, functioning as LTi cells, can secrete IL-17, and IL-22, promoting the stroma to induce expression of CXCL12 and CXCL13 (56, 57). B cells also have been shown to function as LTi cells, secreting IL-22, and regulating TLS formation (49). M1 macrophages, functioning as LTi cells, have also been shown to control TLS formation in colorectal carcinoma by secretion of the pro-inflammatory cytokine IL-36γ (58). M1 macrophages in rat models of chronic graft rejection additionally can produce high levels of LTα and TNF-α, thereby functioning as LTi cells (59). IL-13, another inflammatory cytokine present in the TME, has been implicated in stromal cell regulation, allowing for the development of PDPN^+^ immunofibroblasts that function as LTo for TLS development (60).

Additionally, LIGHT/TNFSF14, a T cell costimulatory molecule, can bind LTβR, and initiate lymphotoxin signaling in stromal cells resulting in lymphangiogenesis (61). High expression of LIGHT with expression of LTβR on target stromal cells in metastatic CM was found to associate with CCL21 expression from the stroma and significant T cell infiltration (62). Along with the lymphotoxin family (LTα_1_β_2_, LTα_3_, and LIGHT), TNF-α, another important cytokine associated with improved therapeutic response in CM (63), is also able to induce CCL21 expression (64). This is notable in CM, as tumor derived TNF-α was found to be secreted by murine B16 melanoma cells (65).

## TLS in Cutaneous Melanoma

The presence of DC-Lamp^+^ DC, CD20^+^ B cells, and CD3^+^ T cells has been traditionally used to define TLS (16, 17). Additionally, transcriptional profiling for specific chemokine signatures has proven successful in discerning TLS in the TME. In CM, the presence of DC-Lamp^+^ DC and a 12-gene chemokine signature in the TME can effectively identify TLS^+^ tumors (16, 66–68).

The presence of high-levels of DC-LAMP^+^ and OX40^+^ lymphocytes in TLS in patients with stage Ia – IIIa CM has been associated with improved patient survival (66). In patients with metastatic melanoma, classic TLS with well-defined T cell and B cell/GC zones tend to be more commonly identified (69). The observation that CM metastases may have more developed TLS is interesting given that metastatic CM is genetically more complex than primary CM in exhibiting a higher TMB (7, 70). Interestingly, Posch et al. (71) reported that the TLS in colorectal carcinoma patients with microsatellite instability-high (MSI-H) tumor burden had higher numbers of “classical” TLS containing GC. Indeed, the higher TMB in metastatic CM has been associated with stronger inflammatory immune responses and patient responsiveness to ICI-based immunotherapy (72).

While the idea that advanced disease with higher TMB may be linked to TLS neogenesis, which is in turn associated with positive therapeutic response to anti-PD-1 and anti-CTLA-4 therapy (33, 73), is speculative, the concept of combining ICI with treatments promoting TLS neogenesis for improved clinical outcome is attractive and empirically testable. In advance of the development of such combination modality protocols, we note that there are currently two clinical trials with the induction of TLS in the TME as a clinical endpoint. One is assessing trustuzumab in the neoadjuvant setting for breast cancer patients (NCT03144947), with the other investigating the efficacy of nivolumab plus relatimab (anti-LAG3 mAb) in soft-tissue sarcoma (NCT04095208).

## Melanoma Mutational Status and TLS Induction

The identification of dominant driver mutations in melanoma initiated the development of successful targeted therapeutics against constitutively activated oncogenes (74, 75). Furthermore, BRAF, KIT, and NRAS mutational status predicts therapeutic response in CM patients (76).

The propensity for CM, a mutationally complex cancer, to form TLS is intriguing. Using the rich mutational data compiled in The Cancer Genome Atlas (TCGA), Thorsson et al. (77) classified CM into immune-oriented cohorts that expressed specific gene expression and immunologic responses. The immune expression biomarkers employed in this study included those predicted to promote TLS formation, including pro-angiogenic genes, high M1 macrophage ratio and high CD8^+^ TIL content (77). These pro-inflammatory immune features associated with notable driver mutations in CM may explain, at least in part, CM patient propensity to form TLS within the TME.

Recent clinical and pre-clinical reports have demonstrated that the loss of DNA repair protein expression is associated with increased emergence of neoantigens in a variety of tumor types and models (78–82). Germano et al. demonstrated accumulated neoantigens and improved immune surveillance in colorectal, breast, and pancreatic mouse cancer cell lines deficient in DNA mismatch repair (MMR-deficient). In fact, MMR-deficient tumors had increased infiltration of CD8^+^ and CD4^+^ T cells when compared to MMR-proficient tumors (78). Green et al. (82) similarly reported increased prevalence of CD8^+^ tumor-infiltrating lymphocytes in patient breast cancer tumors that expressed low levels of the XRCC1, ATM, and BRCA1 proteins involved with DNA damage response.

The tumor-associated inflammatory impact resulting from the loss of DNA repair and increased neoantigen expression presents an exploitable immunotherapeutic opportunity. Defects in DNA mismatch repair proteins may act as novel biomarkers for ICI efficacy, specifically anti-PD-1/PD-L1 immunotherapies (83). In a phase 2 clinical trial (NCT01876511), patients with progressive metastatic CRC with MMR-deficiency had increased immune-related objective response and progression-free survival when compared to patients with progressive metastatic CRC with MMR-proficiency after receiving pembrolizumab immunotherapy (84, 85). Additionally, in a mouse model of CRC, it was reported that anti-PD-l, and anti-CTLA-4 ICI significantly impaired the growth of MMR-deficient tumors compared to MMR-proficient tumors (78). Notably, increased levels of CD8^+^ TILs were found in the MMR-deficient tumors.

**Figure 1:**
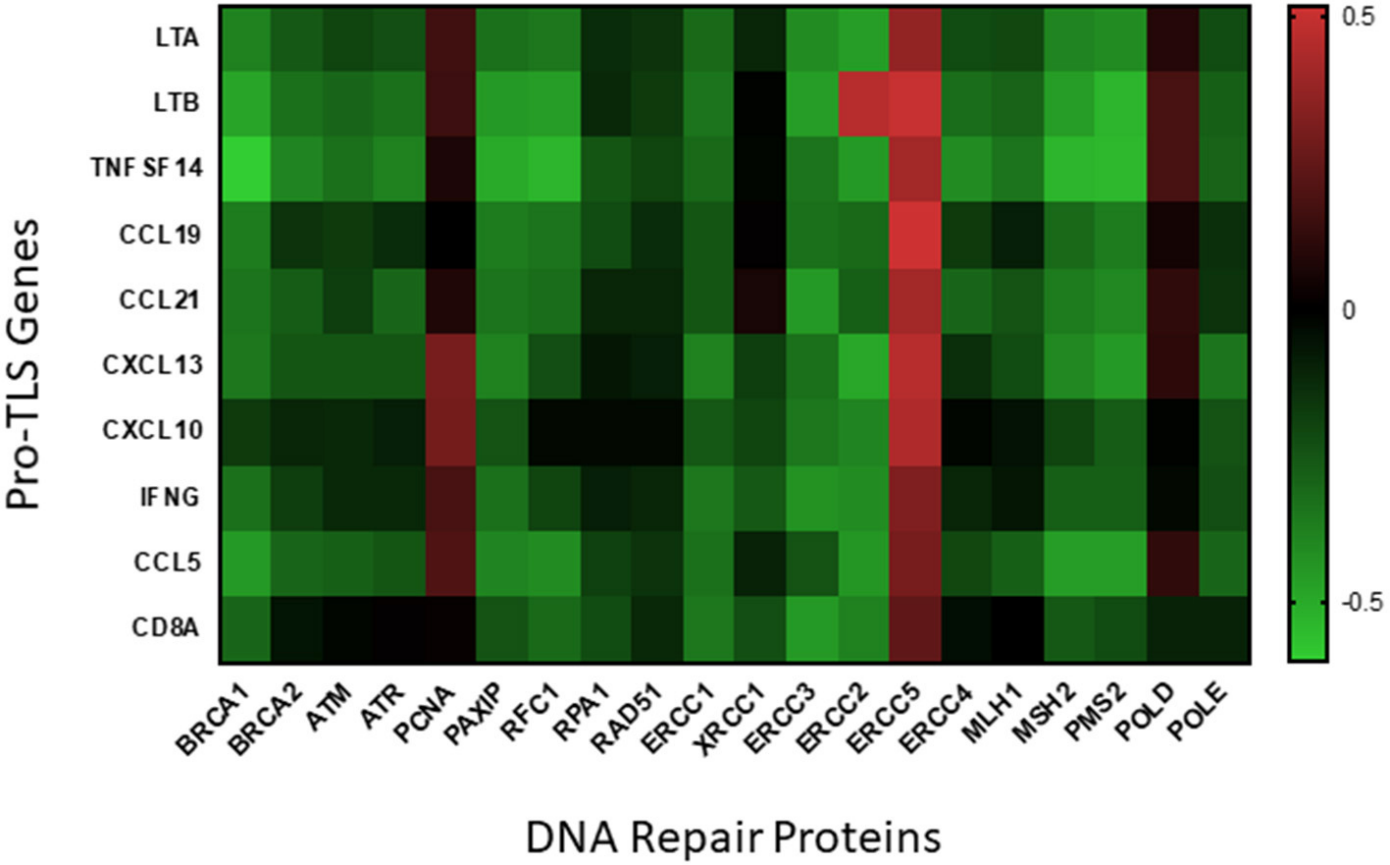
Correlation of DNA repair proteins with pro-TLS genes. TCGA data from two cohorts of cutaneous melanoma patients (SKCM_DFCI 2015 and SKCM_MSKCC 2014) was included to analyze if expression of DNA repair proteins correlated with the expression of inflammatory markers within the TME. TCGA data was uploaded onto cBio Portal, a published TCGA data analysis tool (86, 87), and correlative mRNA expression of the above inflammatory and DNA repair proteins visualized using linear regression models. Pearson's coefficients from each correlation is visualized as a heatmap (created using the Prism 8 software) above with positive correlations indicated in red and negative correlations indicated in green.

The presence of TLS in CM may provide a site for *in situ* neoantigen-specific CD8^+^ T cell clonal expansion, ultimately resulting in an effective antitumor adaptive immune response. Given that tumors with decreased DNA repair pathways may be pre-disposed for increased presence of CD8^+^ T cells, we hypothesize that the TMB of melanoma may be a predictor for TLS neogenesis. By analyzing the genome from two cohorts of cutaneous melanoma patients compiled in the TCGA using cBio Portal, we found that the expression of many DNA repair genes to be negatively correlated with expression of TLS-associated genes (86, 87) (Figure 1). We found a statistically significant inverse correlation between at least eight TLS-associated genes and DNA repair protein elements BRCA1, PAXIP, ERCC1, ERCC2, ERCC3, MSH2, and PMS2. These latter genes transcribe proteins involved in pathways of DNA repair systems including homologous recombination (BRCA1, PAXIP), nucleotide excision repair (ERCC1, ERCC2, ERCC3), and MMR (MSH2, PMS2). Although further in-depth studies are required, these preliminary data appear to support the potential utility of TMB as a predictor for TLS formation in CM.

**Figure 2:**
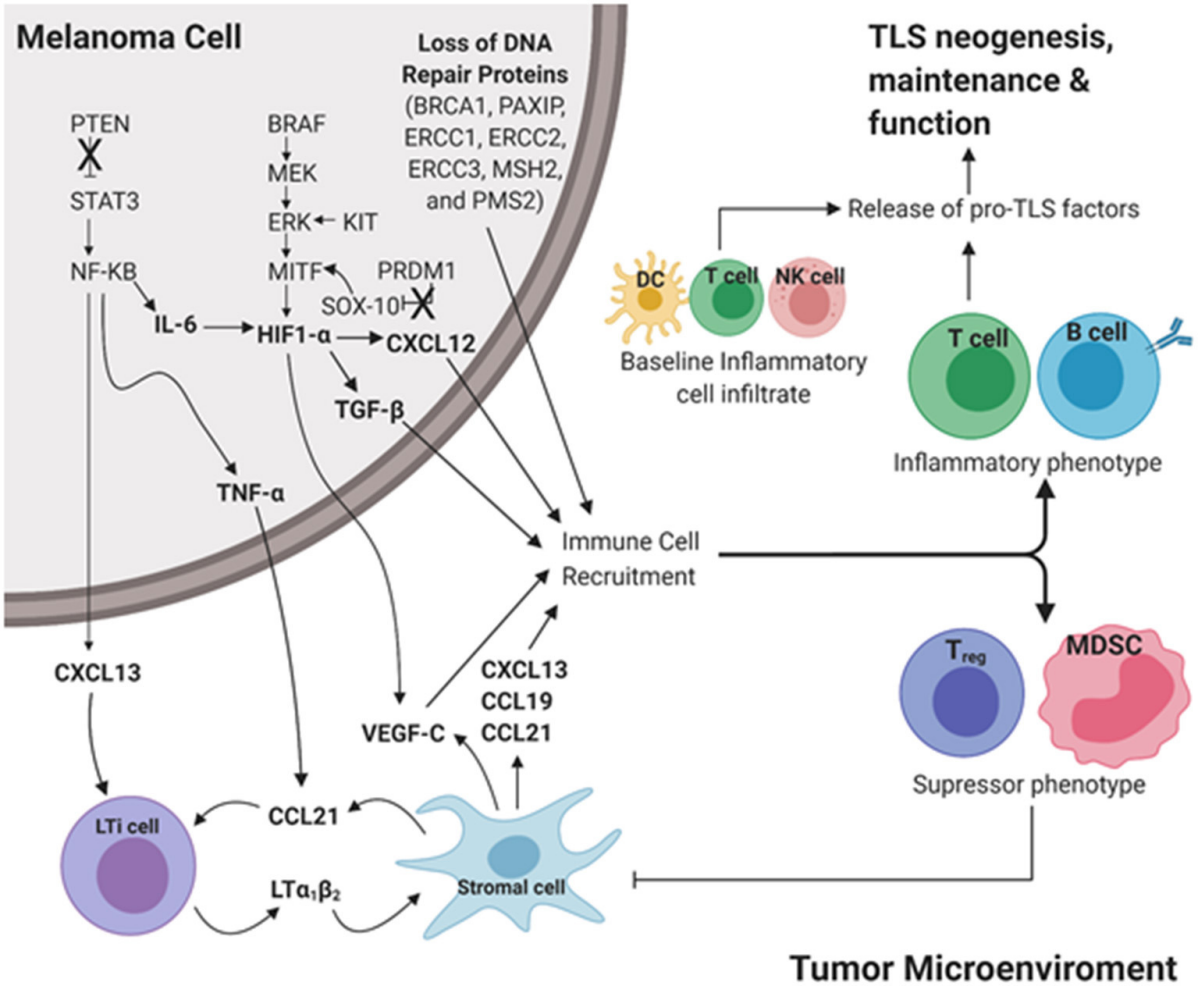
Proposed intrinsic molecular mechanisms that contribute to TLS development downstream of common driver mutations in cutaneous melanoma. Pro-inflammatory cues initiate TLS neogenesis in the peripheral tumor microenvironment (TME). In a melanoma tumor cell, loss of tumor suppressor genes PTEN and PRDM1 results in increased expression of STAT3 and SOX-10, respectively. Gain of function in the proto-oncogenes BRAF, KIT, MITF results in upregulation of MEK, ERK, and HIF-1α, respectively. Culmination of these mutations results in increased expression of pro-TLS cytokines (CCL21, CCL19, CXCL13, LTa1b2) and pro-inflammatory immune cell recruitment (T cell, B cell) contributing to the development, maintenance, and function of TLS in the TME (Inflammatory phenotype). The lack of proinflammatory cues in the TME contributes to the recruitment and maintenance of immunoregulation (Treg, MDSC) resulting in an immunosuppressive TME (Immunosuppressive phenotype). The ever-changing balance of pro-inflammatory vs. regulatory immune function in TLS likely dictates the anti- vs. pro-tumor influence TLS play in disease outcome. LTi cell, Lymphoid tissue inducer cell; MDSC, Myeloid derived suppressor cell; DC, Dendritic cell; NK cell, Natural killer cell. Created with BioRender.com.

Cutaneous melanoma has significantly higher rates of somatic mutations than other cancers. The genes frequently mutated in CM resulting in melanoma driver mutations include *BRAF, MITF, NRAS, KIT, PTEN*, and *PRDM1* (88). Extending these analyses to downstream driver mutations may uncover potential predictors for TLS formation in CM. Here, we briefly review molecular mechanisms downstream of driver mutations for their potential impact on TLS formation in CM (Figure 2).

### MITF

Microphthalmia-associated transcription factor (*MITF*) encodes an important transcription factor for early melanocyte development, and when mutated, functions as a constitutively active oncoprotein to promote tumorigenesis (89). MITF is downstream of the MAPK signaling pathway and also is able to be activated by other transcription factors that can be mutated in CM, such as SRY-box 10 (SOX-10) and cyclic adenosine monophosphate response element-binding protein (CREB) (90).

MITF has been shown to have roles in anti-tumor immunity. MITF knockdown in B16.F10 melanoma cells results in decreased expression of (TLS-promoting) CCL21 and CXCL10 chemokine levels, in association with reduced immune infiltration/inflammation in the TME and enhanced tumor progression (91). MITF is additionally able to upregulate HIF-1α expression by upstream promoter binding of the HIF-1α gene (92, 93). HIF-1α is a potent inducer of VEGF-C, TGF-β, and CXCL12 (93–95) which are known to promote HEV formation and to stimulate LTi cells, both supportive of TLS formation (16, 43, 94).

### BRAF

The proto-oncogene *BRAF* codes for a serine/threonine protein kinase involved in the MAPK pathway (96). *BRAF* mutations are present in 66% of malignant melanoma, with the V600E mutant being the most prevalent (97). The use of FDA-approved targeted BRAFV600E inhibitors (BRAFi) such as dabrafenib and vemurafenib revealed the immunomodulating role of oncogenic BRAF in the TME. BRAFi promote CD4^+^ and CD8^+^ T cell infiltration into human metastatic melanoma in addition to increasing expression of melanocyte differentiation antigens by melanoma cells, leading to enhanced recognition by antigen-specific T cells (98–100). Furthermore, BRAFV600E regulates IL-1α/β transcription in melanocytes leading to upregulation of immunosuppressive genes such as PD-1 ligands and COX-2 in tumor associated fibroblasts (101). BRAF inhibition as well as knockdown of BRAFV600E in multiple patient-derived melanoma cell lines blocked IL-1α production. Although no direct links between BRAFV600E and the production of TLS-associated factors (CCL19, CCL21, CXCL13, LTα/β, or LIGHT) have been reported it stands to reason that BRAFV600E may promote/support TLS formation through indirect mechanisms and/or pathways.

BRAF V600E has been shown to regulate MITF through MEK and ERK mediated phosphorylation of MITF (90). This phosphorylation by ERK can increase MITF *via* BRN2 and simultaneously down-regulate it by targeting MITF for degradation (102). *BRAF* mutations in CM have also been associated with overexpression of the scaffold protein, Grb-2-associated binder 2 (GAB2). GAB2 is a stimulator of HIF-1α, stimulating VEGF-C, and CXCL12, thus inducing HEV formation (103). Interestingly, a retrospective cohort study of patients with stage II or III non-metastatic colorectal adenocarcinoma, reported that tumors with MSI-H and/or BRAF V600E mutation had higher numbers of TLS (defined as CXCL13^+^CD21^+^CD23^+^) (71). Furthermore, the authors observed a significant two-way association between BRAF V600E mutation and MSI-H status.

### NRAS

*NRAS* was the first oncogene to be identified in melanoma (104). Mutations of the *NRAS* GTPase account for 20% of all melanomas and are the second most common driver mutation (105). NRAS G12D positive CM is associated with an overexpression of GAB2, leading to increased HIF-1α, CXCL12, and VEGF-C allowing for TLS development (103).

### KIT

*KIT* (KIT proto-oncogene receptor tyrosine kinase) mutations are found in about 2% of all CM cases (106). Small molecule tyrosine kinase inhibitors (TKIs) have been developed to compete with the ATP-binding site of oncogenic tyrosine kinases. Constitutively active oncogenic KIT is involved in MITF regulation through downstream phosphorylation of ERK, leading to MITF activation (90, 107). Increased MITF expression is subsequently able to upregulate pro-TLS cytokines through HIF-1α (92). Similarly, to *BRAF* and *NRAS* mutations, the upregulation of these downstream effectors from mutated KIT can support TLS formation.

### PTEN

Phosphatase and tensin homolog (*PTEN)* is a commonly mutated tumor-suppressor gene in invasive and metastatic CM. *PTEN* encodes a phosphatidyl-inositol-3,4,5-triphosphate 3-phosphatase protein that is a key regulator of the PI3k signaling pathway and has a role in maintaining anti-tumor immunity (90, 108). In adipose cells, PTEN mutations resulting in loss of function were found to be associated with increased levels of STAT3 (109, 110). Higher levels of STAT3 can activate NF-κB and results in TNF-α upregulation, leading to increased CCL21 expression in support of TLS neogenesis (64, 109).

Increased STAT3 following PTEN loss of function in CM was also found to increase IL-6 levels via NF-κB (111). The increased IL-6 levels can then upregulate VEGF-C, leading to HEV formation and pro-TLS growth (52, 112, 113). Loss of PTEN in a mouse model of prostate cancer lead to NF-κB mediated transcriptional activation of the CXCL13 gene (114). CXCL13 is pivotal in inducing LTi cells, thereby facilitating classical TLS formation (16).

### PRDM1

PRDM1 (PR domain zinc finger protein 1), also known as BLIMP-1, is a tumor-suppressor gene that is an important regulator of neural crest cell (NCC) development and is frequently lost in metastatic melanoma (115). Loss of function in PRDM1 in a zebrafish model of melanoma has been found to result in faster melanoma tumorigenesis and a more aggressive cancer (115). In this same zebrafish model, loss of PRDM1 resulted in increased expression of SRY-box 10 (SOX10), an important nuclear transcription factor involved in NCC progenitor differentiation to melanocytes (115, 116). The increased SOX10 can upregulate MITF expression, resulting in pro-TLS cytokine expression (117).

## Discussion

The mutational genotype of melanoma plays an undeniable role in constitutive tumor immunogenicity and the promotion of intrinsic or therapy-induced anti-tumor immunity. The association of driver mutations with sustained inflammation in TME contributes to fertile soil for the development of TLS in association with superior prognosis and response to interventional immunotherapy. In this regard, we propose that CM, known to exhibit a consensus high TMB, may be predisposed to develop TLS formation, thereby contributing to the classification of CM as an immunogenic disease. Further experimental evidence needs to be conducted to assess the specific role of each driver gene in the regulation of TLS induction and the constitutive/on-treatment operational immune functionality of these lymphoid organs as it relates to patient outcomes and the development of refined treatment designs centered on therapeutic TLS development.

## Author Contributions

DS and RF contributed intellectually, performed literature searches, and prepared/revised the manuscript and figures. MC contributed intellectually, performed data analyses, and prepared figures. WS contributed intellectually, performed data analyses, and revised manuscript and figures. All authors contributed to the article and approved the submitted version.

## Conflict of Interest

The authors declare that the research was conducted in the absence of any commercial or financial relationships that could be construed as a potential conflict of interest.
